# Factors of pre-war educational migration: an investigation of polish medical and dental students in Ukraine

**DOI:** 10.1186/s12909-024-05464-5

**Published:** 2024-05-03

**Authors:** Diana Rokita-Poskart, Anna Koziarska, Aleksandra Ostrowska, Aneta Kucińska-Landwójtowicz, Magdalena Tataruch

**Affiliations:** 1 Department of Regional Policy and Labour Market, Faculty of Economics and Management, Opole University of Technology, Opole, Poland; 2 Department of Internal Medicine and Nephrology, Faculty of Medicine, Opole University, Opole, Poland; 3 Department of Mathematics and IT Applications, Faculty of Production Engineering and Logistic, Opole University of Technology, Opole, Poland; 4 Department of Management and Production Engineering, Faculty of Production Engineering and Logistic, Opole University of Technology, Opole, Poland; 5 Department of Tourism and Recreation, Faculty of Physical Education and Physiotherapy, Opole University of Technology, Opole, Poland

**Keywords:** Medical and dental students, Medicine and dentistry education, Migration of Poles to Ukraine, Educational migration, Factors of migration, Poland, Ukraine

## Abstract

**Background:**

Low admission rates at Polish medical universities led many students to study abroad. Ukraine provided an appealing option for years. The purpose of the study is to investigate the most important factors that motived Polish students enrolled at I. Horbachevsky Ternopil National Medical University in Ukraine to pursue medical and dentistry studies in lower middle-income countries, just before the outbreak of the war. It has become incredibly important to determine crucial economic and non-economic factors.

**Methods:**

The paper draws on a quantitative survey (*N* = 94) conducted among medical and dental students from Poland who had studied at I. Horbachevsky Ternopil National Medical University in Ukraine using a semi-structured questionnaire. To test whether there was a relationship between the importance of the motives, Pearson’s chi-square independence test was employed.

**Results:**

The study found the key economic and non-economic factors for pursuing Ukrainian studies were rejection from Polish tuition-free programs, lower Ukrainian tuition and living costs compared to Poland and to other European Union countries. Determining reasons for choosing Ternopil University were recruiter and friend recommendations and its prestige among Ukrainian medical schools. Gender influenced the ranking of motives.

**Conclusion:**

The article examines the unique pre-war educational migration from Poland to Ukraine – occurring counter to typical flows from lower to higher a lower income country. The study showed that universities should strengthen recruiting efforts and highlight competitive tuition and living costs to attract international students, especially from relatively more developed nations.

**Supplementary Information:**

The online version contains supplementary material available at 10.1186/s12909-024-05464-5.

## Background

Medicine and dentistry are the most popular fields of study in Poland. In the 2022/2023 academic year for one spot in a medical study at a Polish university there were 46 candidates [1]. To satisfy the growing demand for available studies and to prompt a larger availability of health care staff in Poland, the Polish Ministry of Health using a regulation, increases the number of medical and dentistry studies places each year [2]. This initiative is reflected in the authorization of 21 universities in Poland to offer medical programs and 10 universities to offer dental studies in the academic year 2020/2021 [2]. It is important to mention that Poland is one of those countries that record the lowest indicators in the number of practicing physicians and nursing staff per 1000 residents. According to the OECD Raport, there are 2,4 doctors per 1000 residents in Poland and it ranks Poland at the very bottom of European Union member states [3]. The EU average is 3.9, with Austria and Portugal characterized by more than 5 doctors per 1000 residents [3].

Due to their ultra-low acceptance at many universities in Poland, a significant number of future medical and dental entrants are not able to fulfil their educational plans in the country. For many years, essentially until the Russian invasion of Ukraine, an interesting possibility for undertaking medical and dental studies was choosing studies in Ukraine. The education conducted by Ukrainian universities was in English, which enabled Polish students to take a job after graduation not only in Poland but also abroad. Furthermore, after graduating from studies conducted outside of the European Union, Polish medical and dental graduates have to pass a complicated and expensive process of validation of foreign diplomas, in order to follow their profession in the European Union.

Literature studies shows that population migration often occurs in countries a high socio-economic level of development [4]. Ukraine, as a migration destination for Polish students pursing to become doctors, does not meet these criteria. According to 2021 rankings that include GDP per capita as well as the HDI Index, Ukraine ranks lower than other countries from that region − 127 of 266 countries for GDP per capita and 78 of 191 countries for the HDI Index [5]. It granted interest in the motives of studying in Ukraine by Polish students and outlined the importance of economic and non-economic indicators. Mentioned division results from the most common category of indicators determining undertaking study abroad. In understanding the motivations behind academic migration, it is essential to consider both push and pull factors that influence students’ decisions to study abroad [6]. The classic economic indicators included in the push factors category are low socio-economic level of development [7] and weak development perspectives in the country of origin [8]. The key economic indicators considered as pull factors are the development level and the current economic situation in the receiving countries [9], the labour market situation, the level of wages [10, 11], maintenance costs [12, 13] and the level of tuition [14, 15]. A growing body of literature shows that a low level of educational standard in the country of origin and the distance from the receiving country to the country of origin are crucial non-economic factors [7, 9, 11, 12]. The among the non-economic factors determining the undertaking studies abroad, existing family, friendship, community networks and the standard of education in the receiving country are relevant [9, 12, 14–16]. Furthermore, it is imperative to differentiate between temporary and permanent migration, as this distinction further elucidates the underlying motivations and aspirations of migrating individuals [17].

Moreover, growing body of studies has explored the motivation to study medicine and dentistry abroad [18, 19]. Some findings indicate that individual factors are connected to achieving one’s goals, self-development, and willingness to make new friends [20, 21]. Some researchers state the importance of accomplishing the strategy of making one’s childhood dreams come true [22]as well as the desire to travel [23].

Numerous studies on medical and dental students’ migration highlight push and pull factors. Push factors include low educational standards in developing countries, prompting students to seek better opportunities abroad due to poor university recognizability, inappropriate qualifications of professors, and limited educational offer [24]. Additionally, students consider language familiarity, cultural similarity, and economic factors like maintenance costs and tuition fees in the destination country [25–28].

On the other hand, among pull factors, the major driver to undertake medical studies abroad is the better economic and political situation in the destination country [29]. The accessibility to the labour market is crucial, with student migration often serving as the initial step in a strategy to permanently settle and work in the destination country [30]. Pull factors encompass the proximity between the home and destination country, community networks in the emigration country [31–33], the reputation and prestige of foreign universities, and tuition fees.

Despite the large body of the above-mentioned literature, little is known about the factors affecting the decision to undertake medical studies in low- and middle income countries. Recent literature focuses on humanitarian aid, reasons related to the desire to learn old procedure [33]. The desire to improve cultural competence and become more sensitive to the needs of others has also proven to be significant [34, 35]. Among the benefits of studying abroad in low- and middle income countries are the possibility of connecting students with the global community [36, 37].

Taking into account the identified gap in recent literature, the purpose of the study is to investigate the most important factors that motived Polish students enrolled at I. Horbachevsky Ternopil National Medical University to study in lower-middle-income countries like Ukraine, specifically the reason for choosing I. Horbachevsky Ternopil National Medical University. It has become increasingly important to determine crucial economic and non-economic factors and find a relationship between the main factors and individual respondents demographics.

## Methods

### Procedure

The presented study was part of a larger research project involving not only the university in Ternopil but also universities in Lviv, Ivano-Frankivsk, and other institutions with Polish students enrolled in medical and dental programs. However, I. Horbachevsky Ternopil National Medical University was one of the scarce public higher education institutions in Ukraine attracting a significant number of Polish students in the fields of medicine and dentistry. The study was initially scheduled in 2020, but due to the outbreak of the pandemic COVID19, it was finally conducted in 2021. The plan was to conduct surveys using an auditorium technique during classes and labs, for which the rector of I. Horbachevsky Ternopil National Medical University initially gave approval on March 9, 2020. However, due to the need to postpone the survey and pandemic COVID-19 restrictions in 2021, the survey could not be conducted as intended. Finaly, the survey was conducted using a pen-and-pencil interviewing (PAPI) technique on November 11, 2021 during the meeting which was organized by the Dean of International Students Faculty on Polish Independent Day for all Polish students enrolled at the university (153 individuals). While invitation was extended to all 153 individuals, 94 finally attended. Among the attendees, 72 were enrolled in medical studies, while 22 were pursuing dentistry. Despite the distinction between medical and dental fields, both groups were collectively considered due to the resemblance in their educational curricula. They share comparable educational frameworks and encounter analogous challenges and opportunities throughout their academic endeavours. All students present at the meeting voluntary completed the questionnaires, thereby participating in the study. Due to the outbreak of the war in Ukraine, it cannot be continued at the university and other Ukrainian universities where Polish students are enrolled in medical and dental studies.

### Survey questionnaire

The paper draws on quantitative survey (*N* = 94) conducted among medical and dentistry students from Poland who were enrolled at I. Horbachevsky Ternopil National Medical University in Ukraine.

In consideration of the paucity of scholarly attention devoted to the phenomenon of educational migration towards lower middle-income country, an author-conceived survey questionnaire was formulated [38].

The application of the survey and the construction of the questionnaire [38] was methodically informed by extant literature [39–43] and the collective research expertise of the authors in the domain of student migration [44, 45]. The tool was tested in Danylo Halytsky Lviv National Medical University in 2020, just before the outbreak of COVID-19 pandemic. Thus, the final version of questionnaire was presented in Polish and consisted of 10 questions and seven questions about the demographic characteristics. The questionnaire was divided into four parts. In the first section, students were presented with a list of reasons for studying in Ukraine, identified from the literature review, and were asked to evaluate them using a 5-point Likert scale (ranging from ‘not important at all’ with a score of 1, to ‘very important’ with a score of 5). Additionally, they were asked to explain why they chose the university In the second section of the questionnaire students were asked about the plans after graduation (combination of multiple-choice question and free text), expected wages for employment in Poland and abroad and were asked to evaluate in a 5-point Likert scale (from “not important at all”, with score 1, to “very important” with score 5) the list of factors related to staying in Poland as well as their opinion about the shortages of doctors in Poland. The third part of the questionnaire respondents were asked in 5-point Likert scale (from “not important at all”, with score 1, to “very important” with score 5) to estimate the list of push and pull factors for future emigration. The fourth part consisted of seven questions about the demographic characteristics of the respondents.

### Statistical analysis

All calculation were performed using the STATISTICA computer program version 13, serial number: JPZ002E552504ARACD-M. Frequency distributions from a set of descriptive statistical methods were used in this study. This study examined the significance of motives for choosing to study in Ukraine based on four factors: place of residence in the country of origin, age, year of study and gender. Pearson’s chi-square independence test was used to test whether there is a correlation between the significance of the motives (X) and the factor (Y). We considered results to be statistically significant when the *p*-value was less than 0,05 (*p* < 0,05). 

## Results

### Sample characteristics

Among the 94 students participating in the survey the majority were medical students (72) and female (54). A significantly smaller proportion consisted of men (40) and dental students (22). Little more than 19% of the respondents were 19–20 years old, 24.5% were 21–22 years old, 36.2% were 23–24 years old, only 12.8% were 25–26 years old and 7.4% were 27 or more. Among the respondents 19 (21.3%) were the 1st -year students, 11 (11.7%) were second-year students, 16 (17%) were third-year students, 18 (19,2%) were fourth-year students, 20 (21.3%) were fifth-year students and 9 (9.5%) were sixth-year students.

In total 94 students more than 35% came from voivodeships located near the Ukrainian border – 14,89% came from Subcarpathian Voivodeship which is located closest to Ternopil, 11.70% from Masovian and 10.64% from Lublin Voivodeship. A smaller number represented other voivodeships, however three of them, Silesian, Lower Silesian and Lesser Poland were exceptions. The share of respondents from these regions was higher, amounted to 6–9%. The Polish regions are located in the southern part of Poland, which is considerably distant from Ukraine. It appears, however, that this is the result of a good highway and train connections with Ternopil.

### Results of the research about determinants of student migration

As shown in Table 1, the main factors affecting the decision to study medicine and dentistry in Ukraine were lower tuition fees than in other countries (61.7% of respondents indicated as very important and important), the fact that respondents did not get to study in Poland (59.58% of respondents indicated as very important and important), the fact that courses were taught in English (59.58% of respondents indicated as very important and important) and the lower cost of living in Ukraine (46.81% of respondents indicated as very important and important). Three of these motives relate to the country of emigration – pull factors (tuition fees, courses in English and lower cost of living) and one is related to the country of origin – push factor (did not get to study in Poland).

**Table 1:** Factors for studying in Ukraine

	Very important		Important			Neutral			Little importance			Insignificance at all			No answer	
	n	%		n	%		n	%		n	%		n	%		n (%)
Rejected from universities in Poland	49	52.1%		7	7.45%		13	13.8%		7	7.45%		18	19.3%		
Rejected from universities in other countries	8	8.51%		2	2.13%		2	2.13%		8	8.51%		73	77.7%		1(1.06%)
Lower tuition fees than in other countries	43	45.7%		15	16.1%		18	19.2%		7	7.45%		11	11.7%		
Lower living costs than in other countries	25	26.6%		19	20.2%		17	18.1%		13	13.8%		19	20.2%		1 (1.06%)
University prestige	8	8.51%		15	16.0%		18	19.2%		24	25.5%		28	29.8%		1 (1.06%)
desire to study abroad	14	14.9%		12	12.8%		22	23.4%		22	23.4%		22	23.4%		2 (2.14%)
English-lead courses	28	29.8%		28	29.8%		21	22.3%		8	8.51%		9	9.57%		
Recommended by recruiters	19	20.2%		16	17.0%		12	12.8%		13	13.8%		32	34.1%		2 (2.14%)
Recommended by friends who already have been studying at this university	16	17.0%		20	21.3%		11	11.7%		13	13.8%		33	35.1%		1 (1.06%)

The percentage of students who declared that other factors were crucial was lower. Nevertheless, the recommendation by a friend who studied in Ternopil and the recommendation by study recruiters were also vitally important – about 38% of respondents declared that these motives were very important or important.

It was demonstrated that the significance of the selection factors does not depend on the place of residence, age, and year of study. In each of these cases, the calculated chi-square statistic from the sample was not in critical region, thus the null hypothesis of no dependence could not be rejected (Appendix 1). The dependence of selection factor importance on gender showed a similar pattern (Appendix 1), with one exception: the rank of “lower tuition fees than other countries” as a factor depends significantly on gender (Table 2). In this case, the calculated chi-square test statistic fells in the critical region (*p* = 0.014), leading to rejection of the null hypothesis of no dependence. Thus, at a 0.05 significance level, there is evidence that the importance ranking of this particular factor differs by gender. Furthermore, the negative Spearman’s rank correlation coefficient is not statistically important (*p* = 0,175) but it indicates that women ranked lower tuition fees as a more important factor compared to men. Among the surveyed respondents, this factor is considered very important by 56% of women compared to only 32% of men.

**Table 2:** Person’s Chi-square test results

	Chi-square	df	*p*
Pearson Chi-square (*)	12.54793	df = 4	*p* = 0.014
M-L Chi-square (**)	12.87060	df = 4	*p* = 0.012
Spearman rank R (***)	− 0.141112	t=-1.367	*p* = 0.175

(*) Pearson’s Chi-Square Test; (**) Likelihood Ratio Chi-Square Test, (***) Spearman’s Rank Correlation Coefficient

### Reasons for choosing I. Horbachevsky Ternopil National Medical University

Regarding the reasons for choosing at I. Horbachevsky Ternopil National Medical University two of the most important reasons were identified (Table 3) – the recommendation made by other students and recruiters (22.34%) and the university reputation among other medical Ukrainian universities (21.28%). Slightly less important in terms of motivation to study in Ternopil were tuition fees (14.89%) and proximity to Poland (9.57%). Among those whose most important reason was proximity to Poland, most students came from southern regions in Poland – bordering Ukraine (Subcarpathian Voivodeship) or well connected by a highway or railroad with Ternopil. A minority of respondents cited other important reasons for choosing I. Horbachevsky Ternopil National Medical University such as the absence of corruption (7.45%), the city’s appeal (5.32%) and the university website (3.19%). It should be noted that about 17% of the respondents declared other reasons which were not classified in the mentioned groups, including factors such as classes conducted in English or family residing or studying in Ternopil. Additionally, more that 21% did not declare any reasons.

**Table 3:** Factors of studying in I. Horbachevsky Ternopil National Medical University

	*N* = 94	%
Recommendation by friends/recruiters	21	22.3%
Prestige	20	21.3%
Tuition fee	14	14.9%
Close proximity to Poland	9	9.6%
Lack of corruption	7	7.5%
The city	5	5.3%
The website	3	3.2%
Other	16	17.0%
Not indicated	20	21.3%

* Respondent could enter more than one answer

## Discussion

A review of the literature underscores the distinctive nature of pre-war educational migration from Poland to Ukraine. This migration occurred from a country with a higher level of socio-economic development, better labour market prospects, and higher wage rates to a country with poorer economic indicators. Indeed, recent scholarly works predominantly emphasize educational migration in the opposite direction – from countries with lower income levels to those with more advanced economic development and higher wages. This study represents the only research focused on educational migration of Polish students to Ukraine, concentrating specifically on the key group of medical and dental students – the future medical workforce facing shortages in many Central and Eastern European nations [37], particularly Poland. Survey data revealed the most influential factors driving study in Ukraine were typically non-economic and economic reasons. Analysis of very important factors showed over half of respondents (52.13%) indicated that rejection from tuition-free full-time programs in Poland was a major factor. Of slightly lesser but still substantial significance was the economic factor of lower tuition costs in Ukraine versus other countries (45.74%). Notably, some students rejected from full-time Polish medical and dental studies could pursue part-time paid options in Poland, although the tuition fees were there almost double compared to those in Ukraine. Moreover, 46.81% of respondents highlighted that living costs are far higher in Poland than Ukraine.

The presented study addressed to the role of economic factors in undertaking studies in Ukraine, did not find any confirmation in previous studies. A large body of literature indicates that classical economic factors are either difference in socio-economic development [6] or differences in professional development perspectives between the receiving country and the country of origin [7]. Attention should be paid to the reason of undertaking studies abroad, which can be the labour market situation or wages level [10, 11], maintenance costs [12, 13] and tuition fees [14, 15]. It can be stated that more often in studies concentrated on factors for undertaking medical and dental studies abroad than analysing the migration of students in general, maintenance costs and tuition fee in the receiving country are stated [28, 29].

According to the push-pull migration theory presented by Everet Lee [5], the most important factor of students’ migration – rejected from free full-time studies in Poland - should be categorized as a push factor and the second – lower tuition costs in Ukraine compared to other countries – should be categorized as pull factors. Moreover, it should be mentioned that the crucial issue in the educational migration of Polish students to Ukraine is the coexistence of both factors. Presumably, had the first not existed – the students got into the tuition-free full-time studies in Poland, they would have not considered studying in Ukraine and low tuition fees would have not mattered.

While a major body of literature points to differences in the intention to study abroad between men and women [46], it does not identify the significance of individual factors behind these decisions. The results of statistical analysis have shown that gender influences the ranking of migration drivers for women, a lower tuition fee is of greater significance than for men. This is aligned with other research findings related to differences between women and men [46, 47].

The presented studies allowed for the identification of the critical reasons for choosing Ternopil and at I. Horbachevsky Ternopil National Medical University. Regarding institutional theory and migration network theory [48], it has been determined that the most important reason for choosing a place to study was the recommendation by students, graduates, and friends (22.34%). If not for those already existing interpersonal networks as well as recruiters collaborating with the university who supported the students in applying for a visa, collecting documents necessary for primary application, and finding suitable housing and encouraging them to apply to I. Horbachevsky Ternopil National Medical University, the migration towards Ternopil and choosing this university would not have happened.

Studies conducted on reasons for choosing I. Horbachevsky Ternopil National Medical University showed that almost as often as recommendation made by friends and recruiters, respondents pointed out the prestige of the university among other medical higher educational institutions in the country (21.28%). These findings confirm those of other scientific studies on determinants of international students’ migration, in which the prestige of higher education institution plays an important role [12, 32, 43, 49].

Less important when it comes to choosing a university were the tuition fee (14.89% respondents), proximity to Poland (9.57%) and lack of corruption (7.45%). Given the significance of corruption in the Ukrainian tertiary education system [50, 51] our findings indicate that this issue may also play a role in the decision-making process regarding education.

The primary limitation of the study was the fact that the research was conducted at only one university in Ukraine attended by Polish students, resulting in a relatively small sample size for the survey population. However, it has to be noted that presented study was a part of larger research project involving not only the university in Ternopil, but also in Lviv, Ivano-Frankivsk and other universities with having Polish students as a part of medical and dental studies. Unfortunately, firstly because of the outbreak of COVID-19 pandemic and secondly, due to escalation of the conflict, the plans had to be reconsidered [52, 53]. Hence, the results of the study should be regarded as a retrospective examination of only a part of educational migrations from Poland to Ukraine and should serve as a starting point, laying the groundwork for explanatory hypotheses that can later be thoroughly tested in full-fledged quantitative research.

## Conclusions

Our research sheds new light on factors influencing undertaking the study medicine and dentistry in Ukraine by Polish students enrolled at I. Horbachevsky Ternopil National Medical University. Our study found the most influential motivations were rejection from tuition-free Polish universities and lower tuition costs in Ukraine versus Poland and other countries. Additionally, substantially lower living expenses in Ukraine compared to Poland and the EU proved critical. These factors highlight both push and pull factors in educational migration.

Furthermore, the study delves into the motivations behind the selection of I. Horbachevsky Ternopil National Medical University. The key reasons was recommendations from recruiters and friends, along with the institution’s reputation compared to other Ukrainian medical higher education options. Additionally, tuition fees and proximity to Poland played significant roles in students’ decisions.

Our results offer insights for national policymakers and educational institutions contemplating the retention of such crucial students within the country. Gaining an understanding of the multifaceted factors influencing the educational migration process is essential for developing informed policies and mechanisms to support students to stay in the country. This is an exceptionally pressing issue, as our research indicates that only half of the respondents who had migrated to study in Ukraine had intended to return to their home country after graduation. The rest planned to seek employment in other Western European countries and the US [54].

The presented findings should be treated as a retrospective view of the educational migration from Poland to Ukraine. Most Poles studying previously in Ukraine (approximately 600 people) were forced to return to Poland. Law of March 12, 2022 on Assistance to Citizens of Ukraine in Connection with Armed Conflict on the Territory of That Country, enabling Ukrainian citizens, as well as Polish students studying in Ukraine before the outbreak of war, to continue learning at Polish universities. An interesting research topic would be how many Polish citizens continued their studies online at Ukrainian universities and how many managed to transfer to Polish universities. Given the current closure of Ukrainian destinations for both foreign students and Polish students, it would be intriguing to conduct research exploring where Polish students choose to pursue medicine and dentistry education. Investigating how they perceive the challenges of studying in other selected neighbouring countries, such as Slovakia and the Czech Republic, could offer a comprehensive perspective on the factors influencing educational migration.

### Electronic supplementary material

Below is the link to the electronic supplementary material.

Supplementary Material 1

## Data Availability

The datasets are available from the corresponding author on reasonable request.
