# Supplementary material for: Factors of pre-war educational migration: an investigation of polish medical and dental students in Ukraine
Source: BMC Med Educ. 2024 May 3;24:492. doi: 10.1186/s12909-024-05464-5 (PMC11067294; doi:10.1186/s12909-024-05464-5)
Supplement: Supplementary file 1 — Supplementary Material 1 [file 12909_2024_5464_MOESM1_ESM.docx]

Supplementary table 1. Person’s Chi-square result according to the place of residence vs. rejected from universities in Poland

Pearson’s Chi-Square Test; M-L Chi-square – Likelihood Ratio Chi-Square Test; Spearman rank R – Spearman’s Rank Correlation Coefficient

Supplementary table 2. Person’s Chi-square result according to the place of residence vs. rejected from universities in other countries

See supplementary table 1.

Supplementary table 3. Person’s Chi-square result according to the place of residence vs. lower tuition fees than in other countries

See supplementary table 1.

Supplementary table 4. Person’s Chi-square result according to the place of residence vs. lower living costs than in other countries

See supplementary table 1.

Supplementary table 5. Person’s Chi-square result according to the place of residence vs. university prestige

See supplementary table 1.

Supplementary table 6. Person’s Chi-square result according to the place of residence vs. desire to study abroad

See supplementary table 1.

Supplementary table 7. Person’s Chi-square result according to the place of residence vs. English-lead courses

See supplementary table 1.

Supplementary table 8. Person’s Chi-square result according to the place of residence vs. recommended by recruiters

See supplementary table 1.

Supplementary table 9. Person’s Chi-square result according to the place of residence vs. recommended by friends who already have been studying at this university

See supplementary table 1.

Supplementary table 10. Person’s Chi-square result according to year of the study vs. rejected from universities in Poland

See supplementary table 1.

Supplementary table 11. Person’s Chi-square result according to year of the study vs. rejected from universities in other countries

See supplementary table 1.

Supplementary table 12. Person’s Chi-square result according to year of the study vs. lower tuition fees than in other countries

See supplementary table 1.

Supplementary table 13. Person’s Chi-square result according to year of the study vs. lower living costs than in other countries

See supplementary table 1.

Supplementary table 14. Person’s Chi-square result according to year of the study vs. university prestige

See supplementary table 1.

Supplementary table 15. Person’s Chi-square result according to year of the study vs. desire to study abroad

See supplementary table 1.

Supplementary table 16. Person’s Chi-square result according to year of the study vs. English-lead courses

See supplementary table 1.

Supplementary table 17. Person’s Chi-square result according to year of the study vs. recommended by recruiters

See supplementary table 1.

Supplementary table 18. Person’s Chi-square result according to year of the study vs. recommended by friends who already have been studying at this university

See supplementary table 1.

Supplementary table 19. Person’s Chi-square result according to age vs. rejected from universities in Poland

See supplementary table 1.

Supplementary table 20. Person’s Chi-square result according to age vs. rejected from universities in other countries

See supplementary table 1.

Supplementary table 21. Person’s Chi-square result according to age vs. lower tuition fees than in other countries

See supplementary table 1.

Supplementary table 22. Person’s Chi-square result according to age vs. lower living costs than in other countries

See supplementary table 1.

Supplementary table 23. Person’s Chi-square result according to age vs. university prestige

See supplementary table 1.

Supplementary table 24. Person’s Chi-square result according to age vs. desire to study abroad

See supplementary table 1.

Supplementary table 25. Person’s Chi-square result according to age vs. English-lead courses

See supplementary table 1.

Supplementary table 26. Person’s Chi-square result according to age vs. recommended by recruiters

See supplementary table 1.

Supplementary table 27. Person’s Chi-square result according to age vs. recommended by friends who already have been studying at this university

See supplementary table 1.

Supplementary table 28. Person’s Chi-square result according to gender vs. rejected from universities in Poland

****See supplementary table 1.

Supplementary table 29. Person’s Chi-square result according to gender vs. rejected from universities in other countries

****See supplementary table 1.

Supplementary table 30. Person’s Chi-square result according to gender vs. lower tuition fees than in other countries

****See supplementary table 1.

Supplementary table 31. Person’s Chi-square result according to gender vs. lower living costs than in other countries

****See supplementary table 1.

Supplementary table 32. Person’s Chi-square result according to gender vs. university prestige

****See supplementary table 1.

Supplementary table 33. Person’s Chi-square result according to gender vs. desire to study abroad

****See supplementary table 1.

Supplementary table 34. Person’s Chi-square result according to gender vs. English-lead courses

****See supplementary table 1.

Supplementary table 35. Person’s Chi-square result according to gender vs. recommended by recruiters

****See supplementary table 1.

Supplementary table 36. Person’s Chi-square result according to gender vs. recommended by friends who already have been studying at this university

****See Supplementary table 1.
